# Fitness predicts long-term survival after a cardiovascular event: a prospective cohort study

**DOI:** 10.1136/bmjopen-2015-007772

**Published:** 2015-10-22

**Authors:** Martine J Barons, Sally Turner, Nicholas Parsons, Frances Griffiths, Hugh Bethell, Scott Weich, Margaret Thorogood

**Affiliations:** 1 Complexity Science Centre, University of Warwick, Coventry, UK; 2 Basingstoke Cardiac Rehabilitation Centre, Basingstoke, UK; 3 Warwick Medical School, University of Warwick, Coventry, UK; 4 Basingstoke & Alton Cardiac Rehabilitation Centre, Basingstoke, UK

## Abstract

**Objectives:**

To identify the role of fitness, fitness change, body mass index and other factors in predicting long-term (>5 years) survival in patients with coronary heart disease.

**Design:**

Cohort study of patients with coronary heart disease recruited from 1 January 1993 to 31 December 2002, followed up to March 2011 (1 day to 18 years 3 months, mean 10.7 years).

**Setting:**

A community-based National Health Service (NHS) cardiac rehabilitation programme serving the Basingstoke and Alton area in Hampshire, UK.

**Participants:**

An unselected cohort of NHS patients, 2167 men and 547 women aged 28–88 years, who attended the rehabilitation programme following acute myocardial infarction, an episode of angina or revascularisation, and had a baseline fitness test.

**Main outcome measures:**

Cardiovascular mortality and all-cause mortality.

**Results:**

A high level of fitness (VO_2_≥22 mL/kg/min for men, VO_2_≥19 mL/kg/min for women) at completion of the programme was associated with decreased all-cause death, as was a prescription for statins or aspirin, and female gender. Increase in all-cause mortality was associated with higher age and ACE inhibitors prescription. Higher risk of cardiovascular mortality was associated with increasing age, prescriptions for ACE inhibitor, and diagnosis of myocardial infarction or angina as compared with the other diagnoses.

**Conclusions:**

Prior fitness and fitness improvement are strong predictors of long-term survival in patients who have experienced a cardiac event or procedure. Some secondary prevention medications make a significant contribution to reducing all-cause mortality and cardiovascular mortality in these patients. This study supports public health messages promoting fitness for life.

**Strengths and limitations of this study**

- Unselected cohort of patients registered in a National Health Service cardiac rehabilitation centre.
- All data collected by one individual.
- Long-term follow-up of participants; mean 10 years 8 months.
- Effect of missing data examined using multiple imputations.
- Dietary factors, postrehabilitation exercise and medications unknown; height and ethnicity not routinely recorded throughout.

## Introduction

We report the long-term mortality of an unselected cohort of patients who have experienced a coronary event or procedure. While many studies have reported on the effects of treatment and other factors on short-term case fatality,^1–4^ there is little evidence published on the role of fitness, fitness change, depression and anxiety, and other factors which may be associated with the long-term survival of individuals who have experienced a coronary event (eg, myocardial infarction (MI)) or procedure (eg, coronary artery bypass graft (CABG), percutaneous coronary intervention (PCI)).^5–10^ In addition, the relative importance of two modifiable factors, fitness and body mass index (BMI), has yet to be fully explored.

Coronary heart disease remains the leading cause of mortality in the UK, with coronary heart disease accounting for 18% of all deaths in men and 13% in women.^11^ Nevertheless, the UK has been experiencing dramatic falls in death rates from coronary heart disease in recent years^12^ due to the fall in smoking prevalence^13^ and improvements in the treatment, particularly secondary prevention.^14^

In this analysis, we investigated the factors that influenced long-term survival after a coronary event. We were particularly interested in the effects of fitness, body weight and depression.

## Methods

### Setting and participants

We used data collected by one of us (ST) on patients recruited between 1 January 1993 and 31 December 2002 through the Basingstoke and Alton (Hampshire, UK) cardiac rehabilitation programme^15–17^ with follow-up to 30 March 2011. The cohort was unselected, included all National Health Service (NHS) referrals, and participants have now been followed for 1 day to 18 years and 3 months providing 11 871 person-years of follow-up. Recruitment to the cohort was undertaken typically 2 –6 weeks after their index coronary event.^18^ ^19^ NHS patients in the area served by the rehabilitation centre were routinely referred to this programme following an acute MI, episode of unstable angina or revascularisation. The only other inclusion criterion for the study was that the patients had to be registered with the cardiac rehabilitation programme. This resulted in an unselected cohort of 2714 patients.

The phase III programme —offered at Basingstoke and Alton cardiac rehabilitation centre—assessed patients for physical and psychological health at the beginning and at the end of the programme. They began a supervised aerobic exercise class once or twice a week, with home aerobic exercises in between. The supervised sessions comprised circuit training for 40 min, with those patients needing to rest between different aerobic exercises switching to strength and endurance exercises for ‘active recovery’. Patients graduated from the exercise programme when they could complete the circuit without needing active recovery. Besides the exercise programme,^20^ a health education and stress management component was offered to which patients’ spouses or partners were also invited. This component covered relaxation techniques and a health education programme (understanding coronary heart disease, cholesterol, healthy eating, blood pressure, the benefits of regular physical activity, smoking advice, cardiac medications), and stress management.

### Data

Dates and causes of death were provided by the Office for National Statistics. All patients who attended the programme and had baseline fitness measured were included in the primary analysis. Data collected included whether the programme was completed, diagnosis, comorbidity, family history, occupation, date of birth, age, gender, smoking history, resting heart rate, cholesterol level, triglycerides level, postcode and from 1998, height. At both recruitment and on completion records were made of each patient's weight, blood pressure, fitness, anxiety and depression^21^ ^22^ (as measured by Hospital Anxiety and Depression Scale^17^ ^23^), current smoking habit and medications (ACE inhibitor, aspirin, β-blocker, statins).

We calculated changes in weight, fitness, anxiety and depression from the entry and exit values for each individual. Since height was not routinely recorded throughout, BMI was available for only 889 patients; so, for the primary analysis, we categorised baseline weight into under 78 kg, 78–93 kg and over 93 kg for men, and under 68 kg, 68–80 kg and over 80 kg for women; these categories were labelled I, II and III, respectively, for brevity. These approximate to the normal, overweight and obese categories for men and women of average height (176 cm and 164 cm, respectively; British National Formulary) and the average age of this cohort. The index of multiple deprivation was ascertained from postcode, occupation was coded under nine headings (see table 1), and age was categorised into under 50, 50–59, 60–69 and 70 years and over. The Modified D'Hoore comorbidity index is designed to assess non-coronary comorbidity specifically in the outpatient cardiac rehabilitation environment, rather than in the acute setting,^24^ and was calculated for each patient based on the recorded comorbidities.

**Table 1:** Baseline values for patients at recruitment to cardiac rehabilitation programme

	Male		Female		Total	
Number (n)	1320	(86.3%)	209	(13.7%)	1529	(100%)
Mean years of follow-up (SD)	11.3	(3.8)	11.1	(3.6)	11.3	(3.7)
Mean age in years (SD)	61.0	(9.4)	62.9	(9.0)	61.3	(9.4)

	**n**	**Per cent**	**n**	**Per cent**	**n**	**Per cent**

Age (years)						
<50	158	11.9	19	9.1	177	11.5
50–59	405	30.7	50	23.9	455	29.8
60–69	500	37.9	85	40.7	585	38.3
>70	257	19.5	55	26.3	312	20.4
Diagnostic category						
MI	673	51.0	108	51.7	781	51.1
CABG	382	28.9	51	24.4	433	28.4
PCI	124	9.4	22	10.5	146	9.5
MI+PCI	56	4.3	7	3.3	63	4.1
Angina	61	4.6	19	9.1	80	5.2
Other cardiac diagnoses	24	1.8	2	1.0	26	3.8
Smoking history						
Never smoked	347	26.3	93	44.4	440	28.8
Not for >10 years	430	32.6	30	14.4	460	30.1
Not for 1–10 years	56	4.2	9	4.3	65	4.3
Recent quitter	407	30.8	64	30.6	471	30.8
Current smoker	80	6.1	13	6.3	93	6.0
D'Hoore comorbidity score						
None	968	73.3	141	67.5	1109	72.5
1 (least)	150	11.4	22	10.5	172	11.2
2	168	12.7	42	20.1	210	13.7
3	21	1.6	3	1.4	24	1.6
4 (most)	13	1.0	1	0.5	14	1.0
Diagnosis of diabetes	158	12.0	29	13.9	187	12.2
Family history of CHD	613	46.4	115	55.0	728	47.6
Weight at baseline						
I: <78 kg men; <68 kg women	519	39.3	95	45.5	614	40.2
II: 78–93 kg men; 68–80 kg women	565	42.8	69	33.0	634	41.5
III: >93 kg men; >80 kg women	236	17.9	45	21.5	281	18.3
Medications						
ACE inhibitor						
No	665	50.4	88	42.1	753	49.2
Yes	655	49.6	121	57.9	776	50.7
Aspirin						
No	45	3.4	12	5.7	57	3.7
Yes	1275	96.6	197	94.3	1472	96.3
Statin						
No	455	34.5	57	27.3	512	33.5
Yes	865	65.5	152	72.7	1017	66.5
β-blockers						
No	727	55.1	108	51.7	835	54.6
Yes	593	44.9	101	48.3	694	45.4
Occupation						
Managers and senior officials	236	17.9	16	7.6	252	16.5
Professional occupations	143	10.8	11	5.3	154	10.1
Associate professional	145	11.0	25	12.0	170	11.1
Administrative and secretarial	125	9.5	67	32.1	192	12.6
Skilled trade	362	27.4	13	6.2	375	24.5
Personal service	23	1.7	34	11.5	47	3.1
Sales and customer	26	2.0	16	7.6	42	2.7
Process, plant and machines	155	11.7	12	5.7	167	10.9
Elementary occupations	105	8.0	25	12.0	130	8.5
Fitness						
High baseline	588	44.6	53	25.4	641	41.9
Mid baseline	511	38.7	77	36.8	588	38.5
Low baseline	221	16.7	79	37.8	300	19.6
Depression						
Not depressed	1162	88.0	160	76.6	1322	86.5
Borderline	113	8.6	36	17.2	149	9.7
Depressed	45	3.4	13	6.2	58	3.8
Anxiety						
Not anxious	930	70.5	122	58.4	1052	68.8
Borderline	251	19.0	42	20.1	293	19.2
Anxious	139	10.5	45	21.5	184	12.0
Median VO_2_ mL/kg/min (10th, 90th centile)	21.0	(13.1, 29.7)	15.5	(8.4, 24.5)	20.1	(11.0, 29.2)

CABG, coronary artery bypass graft; CHD, coronary heart disease; MI, myocardial infarction; PCI, percutaneous coronary intervention.

Up to August 1995, fitness was assessed on a bicycle ergometer with ECG monitoring and measurement of estimated peak workload. After that date, exercise tests were performed on a treadmill—without using a handrail, using either the Bruce protocol^25^ or, the modified Bruce protocol, for frail or elderly patients.^26^ Peak exercise tolerance was expressed as the predicted oxygen uptake (VO_2_peak) in mL/kg/min, from the known oxygen cost of bicycling at different workloads.^27^ The same end points were used as for bicycle tests and VO_2_peak predicted on the assumption that each 1 min of the Bruce protocol uses 1 MET (metabolic equivalent—or 3.5 mL O_2_/kg/min), and that the first three stages of the modified Bruce protocol each use 1 MET. VO_2_peak<15 mL/kg/min was categorised as low fitness, VO_2_peak>22 mL/kg/min as high fitness, and the in-between readings as medium fitness for males (categories established for cardiac rehabilitation by Kavanagh *et al*^28^ ^29^); for females VO_2_peak<13 mL/kg/min was categorised as low fitness, VO_2_peak>19 mL/kg/min as high fitness,and the in-between values were categorisedas medium.^28^ ^30^

Depression and anxiety were categorised into none, borderline and depressed or anxious using a Hospital Anxiety and Depression Scale score below 8 to suggest no depression or anxiety, 8–10 to suggest borderline, and a score over 10 to suggest clinical depression or clinical anxiety.^23^

We are confident that all deaths in UK have been captured and the loss to follow-up will have been minimal, perhaps due to deaths which occurred abroad and were not reported to the study.

In the main analysis, baseline categories of fitness, depression and anxiety were used as predictors. In order to test whether an improvement in fitness, depression and anxiety categories influenced survival, and whether these depend on baseline categories, we defined variables fitness, anxiety and depression where a scale of 1–7 captured each starting category and the improvement or deterioration (see online supplementary table S6, which details the categories). Very few patients started in the highest fitness category and then deteriorated over the course of the programme (5 men); so we combined this category with the ‘no change’ category. There were no patients who deteriorated after having started in the mid-fitness category; therefore, we omitted this category and were finally left with five categories. We defined survival time as the period between the date the participant joined the programme and the date of death. In the survival analyses, we considered all-cause mortality and cardiovascular mortality, with non-cardiovascular deaths treated as censored in the latter analysis.

### Statistical analysis

We used Cox proportional hazards models^31^ starting with the subset of variables that were found to be significant predictors (at 5% level) of all-cause mortality in the univariate analyses. We employed a backward stepwise selection algorithm to find the model with minimum Akaike Information Criterion (AIC),^32^ retaining age and gender as the minimum model. All analyses were carried out in R statistical software.^33^ The primary model (model A) was based on 1529 cases with complete data, including a baseline fitness test. Two secondary models were also produced. One used the 1029 cases with complete data—including a baseline and end fitness test—to study the effects of fitness change (model B) on long-term survival. The other used the 889 cases with a baseline fitness test and a baseline BMI measurement to evaluate the relative effect of BMI and fitness (model C) on long-term survival.

Since there was a large amount of missing data, we assessed the credibility of the complete-case model. Analysis of a complete-case data set alone cannot be expected to give reliable estimates of model coefficients without making assumptions about the missing data.^34^ We compared HRs from the complete-case analysis with those from an analysis of all data after replacement of missing values with imputed data. Multiple imputation was performed in the R package MICE (Multivariate Imputation using Chained Equations)^33^ providing 20 completed data sets with values imputed where these were missing. The optimised model was then built with each of these 20 data sets, and pooled estimates of model coefficients and variances calculated using the rules devised by Little and Rubin^34^ for comparison. There were no known reasons to believe that the data were missing other than at random, except in the case of the end fitness data. Reasons for end fitness to be missing included referral for cardiac surgery and poor health; a Student t test confirmed that time to death for these individuals differed from those with end fitness observations.

## Results

### Patient characteristics

Of the 2964 patients with coronary heart disease recorded on the database, 160 did not attend any part of the rehabilitation programme, 1 of them had a MI suspected to be secondary to anorexia nervosa, another was 18-year-old probably a miscode, and 88 did not have a fitness assessment at entry to the programme; this left 2714 patients. A total of 2054 patients completed the programme^17^; the main reasons for not completing the programme were patient preference, referral for cardiac surgery, poor health or death. Those without complete end fitness data included the ones who were taken to a different heart rate at the final fitness test, most of whom were on a β-blocker for which the dose had been changed or who were tested at a different time of day, and those who were tested using a different test protocol.^17^ The mean follow-up for those with complete data was 11 years 4 months.

**Figure 1:**
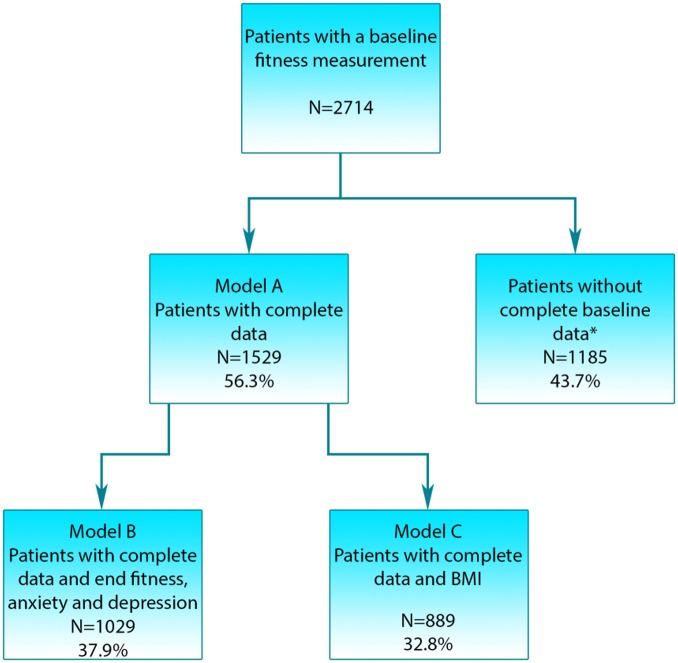
The number of patients* with missing data in individual variables of interest varied, with fitness after the programme missing in 48.5% of cases, and the remaining variables missing data ranging between 0% and 29.4%. Details of variables and numbers, and percentage of missing observations are provided in online supplementary table S1 (BMI, body mass index).

There were 1529 cases (56.3%) which were complete in all the 36 variables that were significant in the univariate analysis and became the starting point for the primary analysis (model A), see figure 1. Patient characteristics for these are given in table 1. There were 1029 cases (38%) which were complete in the variables used in the primary analysis and also had observations for fitness, depression and anxiety at the end of the programme, allowing us to assess the impact of changes in these variables on survival (model B). Patient characteristics for these are given in online supplementary table S5. There were 889 cases (32.8%) for which it was possible to calculate BMI and were complete in the other variables (model C). Patient characteristics for these are given in online supplementary table S7.

Women (13.7%) had a higher mean age. The most common reason for referral to the programme was acute MI (51.1%). Nearly half the women had never smoked compared with just under one-third of the men, and close to one-third of both genders had recently given up smoking. Around half of all patients had a family history of coronary heart disease and over 70% were free from non-coronary comorbidity, although a higher proportion of the women had diabetes. The men had a higher mean fitness than the women, and the percentage of men in the high-fitness category at recruitment was twice that of the women. At completion, 67% of the men and 41% of the women were in the high-fitness category, with one-fifth of the patients having improved from the mid-fitness category. Median fitness was improved by a similar amount in both genders, and high fitness was the largest group at completion.

There was little evidence of clinical depression in this cohort, and most of those whose scores suggested a borderline category improved by programme completion (83%), as did almost all of the few whose scores at recruitment suggested clinical depression (83%). There was more anxiety, although the rates were not high. Again, the majority who began in the borderline category improved, as did a significant proportion of those starting in the clinical anxiety category, but the proportion of women who remained in the clinical anxiety class was three times that of the men. The maximum recorded comorbidity score was 7 (very high), but only 2.6% of participants had a comorbidity score of 3 or more (see online supplementary table S2 for scoring details).^24^

### Survival

During the course of the study and follow-up, 385 participants (25.2%) died and of these deaths, 192 (49.9%) were from cardiovascular causes.

For model A, the primary proportional hazards model, the significant predictors of all-cause mortality are presented in table 2. In this model, age was the most important predictor of all-cause mortality, with risk of death increasing with age. The next most important predictor was fitness category at recruitment, with the fittest patients having the lowest risk. Risk increased with comorbidity, and aspirin and statins prescriptions reduced risk. MI was the diagnosis carrying the greatest risk, including MI with PCI, angina and other cardiac diagnoses. CABG and PCI alone were much lower risk diagnoses. Those with a lower resting heart rate were at a lower risk of mortality. Higher systolic blood pressure was also a risk indicator. ACE inhibitor prescription was associated with higher risk; the most likely reason for this is that at the time these drugs were being prescribed the ACE inhibitors were only given to individuals at high risk. That is, the users of ACE inhibitors will have been at higher risk than non-users before starting to use the medication. After adjusting for other factors, gender was not a statistically significant predictor of mortality. The significant terms from the cardiovascular mortality model are detailed in table 3. Fitness category at baseline was the strongest predictor of cardiovascular mortality, with higher fitness associated with lower risk. A prescription for statins cut the risk of cardiovascular mortality by more than half in this cohort. Age was the next most significant predictor of mortality, with risk increasing with age as expected. As with all-cause mortality, a diagnosis of MI carried the highest associated risk of mortality, with MI+PCI, angina and other cardiac diagnoses having an equally high mortality risk. A prescription for ACE inhibitor was associated with higher risk.

**Table 2:** All-cause survival model A (using only baseline fitness, anxiety and depression categories), ordered by importance of variables to the model (1529 cases, 385 deaths)

	Complete cases			Imputed data
		95% CI		
Model term	HR	Lower	Upper	HR
Age category(years)				
<50	1.00	–	–	1.00
50–59	2.13	1.17	3.88	2.04
60–69	3.85	2.15	6.89	3.09
>70	7.96	4.38	14.44	6.12
Fitness at baseline				
High baseline	1.00	–	–	1.00
Mid baseline	1.54	1.16	2.05	1.75
Low baseline	2.47	1.78	3.42	2.67
D'Hoore comorbidity				
None	1.00	–	–	1.00
1 (least)	1.23	0.91	1.66	1.74
2	1.42	1.08	1.86	1.40
3	1.35	0.68	2.66	1.96
4 (most)	4.50	2.13	9.50	2.09
Statins				
Yes	0.72	0.57	0.89	0.74
No	1.00	–	–	1.00
Aspirin				
Yes	0.52	0.35	0.78	0.63
No	1.00	–	–	
Systolic blood pressure before: each 1 mm Hg reduction	0.995	0.993	0.999	0.998
ACE inhibitor				
Yes	1.26	1.01	1.57	1.18
No	1.00	–	–	1.00
Resting heart rate	1.007	1.000	1.014	1.007
Diagnostic category				
MI	1.00	–	–	1.00
CABG	0.69	0.54	0.89	0.69
PCI	0.53	0.32	0.88	0.67
MI+PCI	0.84	0.44	1.65	0.79
Angina	0.87	0.54	1.39	0.84
Other cardiac diagnoses	0.99	0.44	2.25	0.93
Gender				
Male	1.0	–	–	1.00
Female	0.79	0.59	1.06	0.69

CABG, coronary artery bypass graft; MI, myocardial infarction; PCI, percutaneous coronary intervention.

**Table 3:** Optimised cardiovascular survival model A (using only baseline fitness, anxiety and depression categories), ordered by importance of variables to the model (1529 cases, 192 cardiovascular deaths)

Model term	Complete cases			Imputed data
		95% CI		
	HR	Lower	Upper	HR
Fitness				
High baseline	1.00	–	–	1.00
Mid baseline	1.83	1.20	2.79	2.34
Low baseline	4.06	2.58	6.39	4.26
Statin				
Yes	0.45	0.33	0.61	0.51
No	1.00	–	–	
Age (years)				
<50	1.00	–	–	1.00
50–59	1.58	0.76	3.31	1.53
60–69	2.63	1.30	5.35	2.39
>70	4.00	1.93	8.28	3.77
Diagnostic category				
MI	1.00	–	–	1.00
CABG	0.60	0.42	0.85	0.62
PCI	0.26	0.09	0.70	0.49
MI+PCI	1.05	0.42	2.62	0.74
Angina	0.85	0.46	1.53	0.72
Other cardiac diagnoses	0.79	0.25	2.49	0.99
Aspirin				
Yes	0.47	0.28	0.78	0.57
No	1.00	–	–	1.00
Gender				
Male	1.00	–	–	1.00
Female	0.73	0.48	1.10	0.62
ACE inhibitor				
Yes	1.42	1.04	1.94	1.36
No	1.00	–	–	1.00

CABG, coronary artery bypass graft; MI, myocardial infarction; PCI, percutaneous coronary intervention.

The all-cause mortality for model B, the cohort having complete data including end depression anxiety and fitness, is detailed in online supplementary table S3. In model B, age was the strongest predictor of risk for all-cause mortality, followed by the combination of fitness category at recruitment, and whether the patient improved or maintained that fitness, with highest risk attributed to those who began in the low-fitness category. Mean VO_2_peak change was 3.81 VO_2_ mL/kg/min (SD 3.38). There was no statistically significant difference (assessed at the 5% level) between those who began in the mid-fitness category and improved to high fitness and those who began in the high-fitness category and maintained high fitness. However, those who did not improve sufficiently to move up from the mid-fitness category had significantly higher risk; improvement to a mid-fitness from low-fitness category did not significantly reduce risk, although a significant difference in risk is evident between low and medium fitness for the patients whose category did not change. Having a prescription for statins or having a prescription for aspirin was each associated with a lower risk of mortality. A prescription for ACE inhibitors was associated with a higher risk of mortality.

The cardiovascular mortality for model B is detailed in online supplementary table S4. Fitness was the most powerful predictor of cardiovascular mortality, low baseline and failure to improve being powerful predictors of cardiovascular mortality. A prescription for statins was important, with those having a prescription having one-third the risk of those without. After fitness, age was the most important factor, with those over 70 years at higher risk of cardiovascular death. Aspirin was associated with lower risk of mortality.

The all-cause and cardiovascular mortality for model C, with BMI as a prognostic factor, is detailed in online supplementary tables S8 and S9. Model C was very similar to the primary model. Notably, while BMI was a predictor of mortality in the univariate analysis, in the presence of a fitness assessment it ceased to be statistically significant. This suggests that an assessment of fitness may be a better prognosis of survival than a measure of obesity in people with coronary disease.

### Imputed data

The HRs derived from the pooled imputed data (shown in tables 2 and 3) are reassuringly very similar to those from the complete-cases model, both in size and direction, indicating that missing data has not significantly modified the inferences we report.

## Discussion

We set out to discover which factors predict mortality in an unselected cohort of patients attending cardiac rehabilitation. We found that having a good level of fitness before a coronary event or procedure confers a survival advantage. Also, individuals who increase fitness during cardiac rehabilitation could increase life expectancy, even if they were already moderately fit.

Four major strengths of this study are that there was a long follow-up period, the participants were unselected, fitness was assessed at entry into rehabilitation, and all the data were collected in routine clinical practice by one physiotherapist (ST) using a consistent fitness delivery programme. The data reported here are derived from a single NHS centre, with a consistent delivery of the programme spanning 18 years (average follow-up 11.5 years). Since all patients registered in the programme with a baseline fitness test were included, the participants represent a realistic sample of the population of people who are likely to access cardiac rehabilitation.

The majority of trials of cardiac rehabilitation have followed participants for 2 years or less,^17^ and of the studies that have reported on long-term survival, very few consider fitness as a possible explanatory variable.^13^ ^14^ ^35^ However, a recent Canadian study of a similar duration to ours, but which followed a selected cohort of patients,^30^ also found that baseline fitness and fitness improvement were associated with reduced all-cause mortality. The authors selected a group of patients who had undergone cardiac catherisation procedures, had completed a 12-week exercise-based cardiac rehabilitation course, had returned 12 weeks later for a repeated assessment, and had survived for at least 6 months from the date of catherisation. There were no missing data. The study participants were of a similar age range and gender balance as those in our study, but their baseline fitness was higher.

Our findings were based on routinely collected clinical data in an unselected group of patients, with inevitably some missing data. However, we have been able to demonstrate the robustness of our models using established statistical methods. Physical fitness was assessed indirectly using predicted, not measured, oxygen uptake. Over the course of the study, two different methods of estimating fitness were used. However, we ensured that only patients whose fitness was assessed by the same method at the beginning and the end of cardiac rehabilitation programme were included in the variable measuring change in fitness. Furthermore, method of fitness assessment was not statistically significant in the survival models. The methods of fitness assessment are typical of the pragmatic methods used in such NHS settings.^36^ Patients who did not have a baseline measure of fitness were excluded, diminishing the generalisability of the results. Some of these individuals will have been the frailest patients. Lower VO_2_peak estimated from a treadmill test can indicate more severe cardiovascular disease as well as lower fitness, and it may be that those patients with low fitness also have more severe cardiovascular disease.

Attaining a fitness level of VO_2_≥22 mL/kg/min for men and VO_2_≥19 mL/kg/min for women in the early months following a cardiac event or procedure is associated with improved long-term survival. For comparison, a man aged 60+ with a VO_2_peak of 26.1–32.2 mL/kg/min is considered reasonably fit and 32.3–36.4 mL/kg/min, as very fit;^37^ so these fitness categories are not very demanding. High fitness at recruitment to the rehabilitation programme was likely to reflect high fitness before a coronary event or procedure, but there was no statistically significant difference between patients who improved from moderate fitness at recruitment to high fitness at programme completion and those who maintained high fitness from recruitment. Since we have followed these patients for a mean of 10.7 years, and have no information on what their levels of exercise or fitness were during those years, it is even more striking that a brief snapshot of their past fitness should show such a strong effect on long-term survival. If long-term data on fitness were available, it may be that the effect would prove to be even stronger. Measured VO_2_peak was found to be a predictor of long-term mortality of men and women in Canada in the earlier decades, without consideration of change in fitness; even moderate fitness conferred a 50% reduction in cardiac mortality.^28^ ^29^

Having adjusted for fitness, BMI does not appear to affect all-cause or cardiovascular mortality in patients with cardiovascular disease, which is consistent with other studies.^10^ ^30^ ^38–40^ These findings are in line with those of a recent large European cohort study which examined reported physical activity and measures of obesity as predictors of all-cause mortality, and concluded that physical inactivity ‘is responsible for more than twice as many deaths as general obesity’.^40^

Using medications for secondary prevention, particularly aspirin and statins, in the early weeks after a cardiac event or procedure appears to reduce long-term mortality from cardiovascular causes and all-causes. This confirms previous findings.^14^ Given that we have no information on adherence or changes to medication after completion of the programme, the strong effect of secondary preventative medication is striking. Analysis of data sets with information on the long-term use of medications might show an even stronger effect. Preoperative risk factors which are good predictors of short-term outcomes have been found to contribute little information to the prediction of long-term survival in patients with CABG.^7^ Traditional predictors of early survival in patients with CABG over the age of 65 years do not affect long-term survival,^10^ but late mortality is increasingly associated with chronic diseases and health behaviours. Long-term prognosis after hospital admission for MI is improving.^41^ In the majority of previous studies, differences between cardiovascular mortality and all-cause mortality were not considered, and nor were secondary prevention medications, fitness or anxiety and depression considered as predictors of survival.

**Figure 2:**
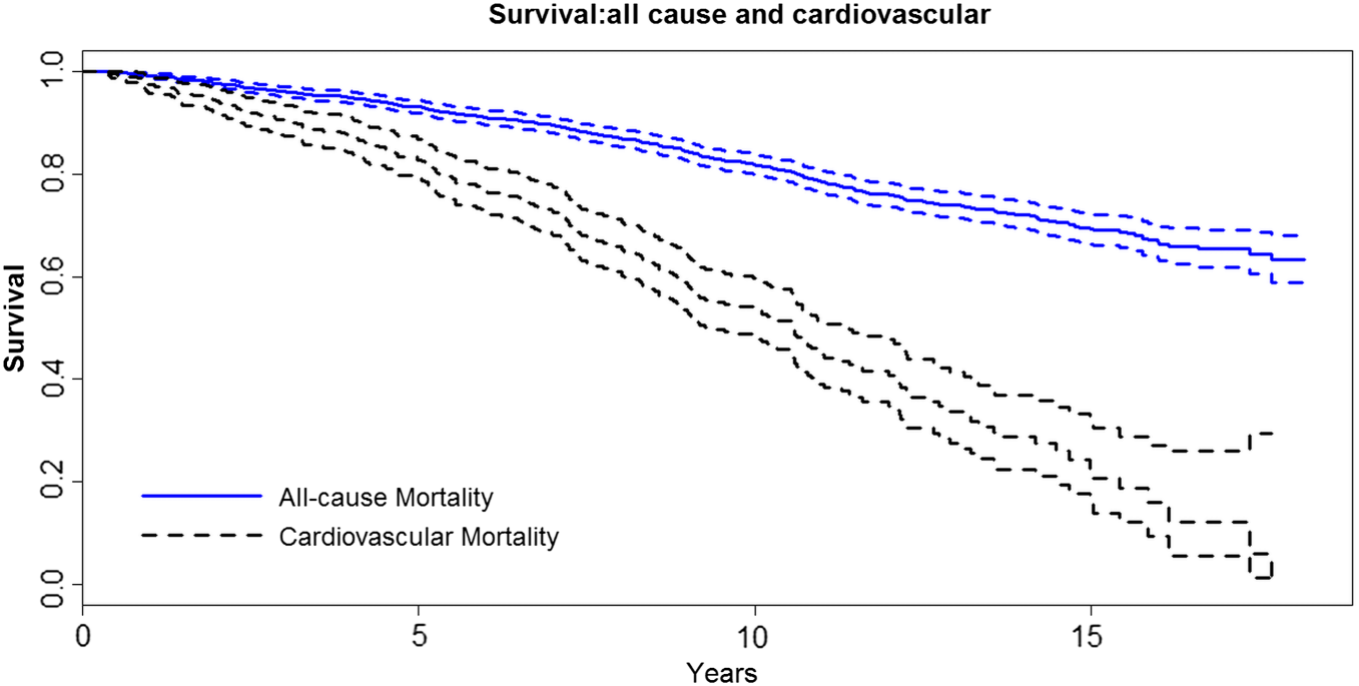
Kaplan-Meier survival curves for all-cause and cardiovascular mortality. The plot is for the entire observation period and the dashed lines are 95% CIs.

**Figure 3:**
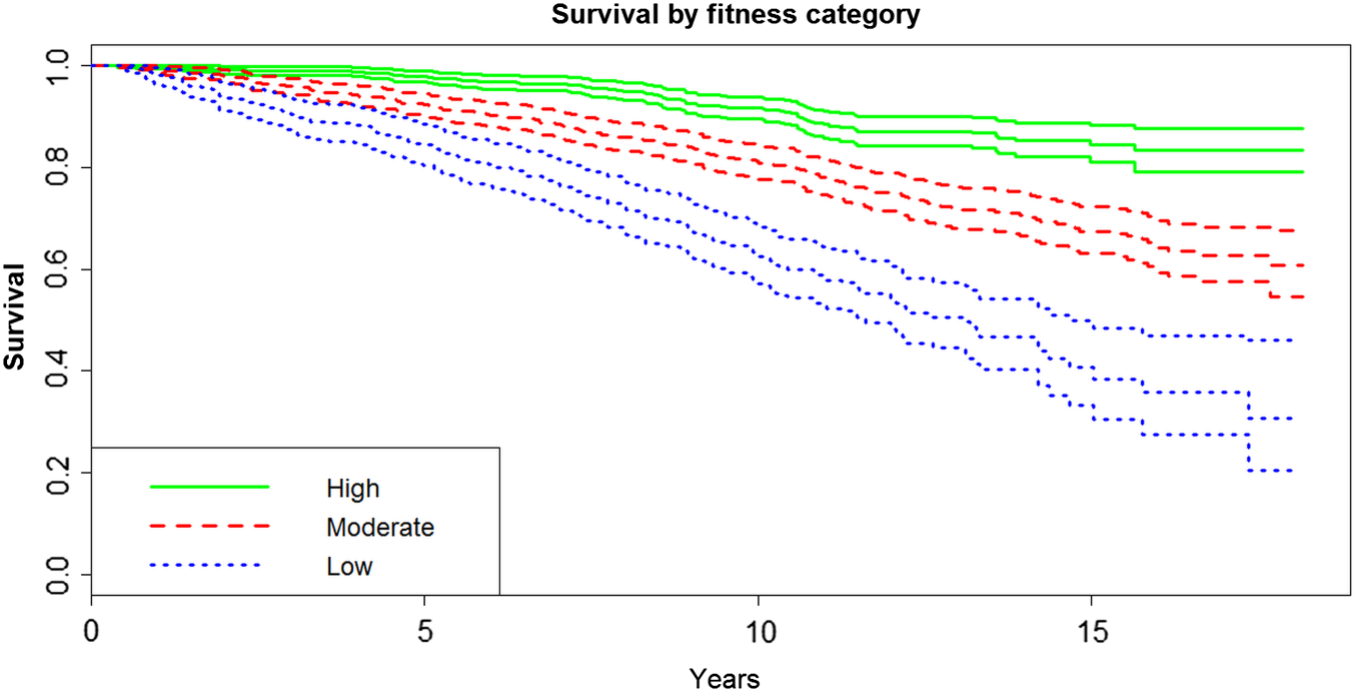
Kaplan-Meier survival curves for all-cause and cardiovascular mortality by baseline fitness group. The plot is for the entire observation period with 95% CIs.

Health may be expected to be generally better in an affluent area, such as the one selected for this study, than in a deprived geographical area; thus these results may not be fully generalisable to other settings. Patients who never attended or attended but did not have a baseline assessment of fitness are excluded from this analysis, but they may form part of the population of patients typically experiencing a cardiac event or procedure, potentially diminishing the generalisability of the results. Some of these individuals will have been the frailest patients, and excluding them also explains the initial period of no deaths seen in the Kaplan-Meier plot (figures 2 and 3). Other limitations include unknown dietary factors, postrehabilitation exercise and medications.

However, our study shows that fitness level in the early weeks after a cardiac event or procedure was equally important for cohorts with a wider range of initial fitness than previous studies, a wider age range, those having significant comorbidities and including women. This analysis contributes to the body of research seeking to identify risk factors for long-term mortality in patients with coronary heart disease.

## Conclusions

Pre-existing fitness or improvement in fitness within the early months after a coronary event or procedure predicts long-term survival of patients. Fitness interventions should take precedence over weight loss interventions.

Secondary prevention medications contribute significantly to improved long-term survival in all-cause and cardiovascular mortality even when there is no information on how long the medications were used, suggesting that the effect is very strong. Patients having coronary artery bypass surgery and PCI have a significantly higher long-term survival from cardiovascular mortality than do patients with a MI or angina. Promotion of fitness after a coronary event or procedure may extend life expectancy.
